# Coexisting Subdural Hematoma in Cerebral Amyloid Angiopathy: A Case Series

**DOI:** 10.3390/neurolint17080125

**Published:** 2025-08-07

**Authors:** Matija Zupan, Lara Straus, Tomaž Velnar, Matic Bošnjak, Ulf Jensen-Kondering, Bruno Splavski, Senta Frol

**Affiliations:** 1 Department of Vascular Neurology, University Medical Centre Ljubljana, 1000 Ljubljana, Slovenia; matija.zupan@kclj.si (M.Z.); lara.straus@kclj.si (L.S.); 2 Faculty of Medicine, University of Ljubljana, 1000 Ljubljana, Slovenia; matic.bosnjak@mf.uni-lj.si; 3 Department of Neurosurgery, University Medical Centre Ljubljana, 1000 Ljubljana, Slovenia; tomaz.velnar@kclj.si; 4 Institute of Pathology, Faculty of Medicine, University of Ljubljana, 1000 Ljubljana, Slovenia; 5 Department of Neuroradiology UKSH, Campus Lübeck, 23538 Lübeck, Germany; ulf.jensen-kondering@uksh.de; 6 Department of Neurosurgery, Dubrovnik General Hospital, 20000 Dubrovnik, Croatia; splavuno@gmail.com; 7 Faculty of Applied Health Sciences, University of Zagreb, 10000 Zagreb, Croatia

**Keywords:** cerebral amyloid angiopathy, neurosurgery, subdural hematoma

## Abstract

Background: Cerebral amyloid angiopathy (CAA) is a common cause of spontaneous intracerebral hemorrhage (ICH) in elderly individuals, and it is characterized by the deposition of amyloid β protein (Aß) in the walls of small-caliber cortical and leptomeningeal vessels. The diagnostic criteria for CAA highlight its association with spontaneous lobar hemorrhage, convexity subarachnoid hemorrhage (SAH), and cortical superficial siderosis but not with subdural hematoma (SDH). This article presents a three-patient case series of CAA who experienced a lobar ICH associated with an SDH, underscoring a potentially under-recognized correlation between an acute ICH and coexistent SDH. Case presentation: We present a case series of three patients in a single university medical center who experienced acute-onset lobar ICH with a concurrent SDH, treated with evacuation. Histopathological examination established the diagnosis of CAA in all three cases. This case series underscores a potentially under-recognized association between an acute ICH and coexistent SDH in the context of CAA. Conclusions: Considering our findings, we emphasize the possibility that SDH may be a more frequent manifestation of CAA than previously recognized. Therefore, patients with CAA who initially present with acute SDH may be underdiagnosed, consequently leading to delayed identification and missed opportunities for proper risk assessment and management.

## 1. Introduction

Cerebral amyloid angiopathy (CAA) is a common cause of spontaneous intracerebral hemorrhage (ICH) in elderly individuals [1]. It is characterized by the deposition of amyloid-β protein (Aß) in the walls of small-caliber cortical arterial vessels, capillaries, and leptomeningeal arteries [2]. The Boston criteria (Version 2.0) for the diagnosis of CAA highlight the association of the condition with spontaneous lobar hemorrhage, convexity subarachnoid hemorrhage (SAH), and cortical superficial siderosis [3]. Nevertheless, these criteria do not include the presence of subdural hematoma (SDH). A definite diagnosis of CAA requires histopathological confirmation from brain tissue obtained at autopsy. However, in clinical practice, CAA diagnosed through biopsy is classified as probable CAA with supporting pathology owing to the potential for sampling error and false-negative results [4].

Several studies have noted the association of non-traumatic SDH with the acute-onset lobar ICH in patients with CAA, which can be observed in approximately 20% of cases [5]. A retrospective cohort study utilizing two large population-based cohorts reported that CAA may be an independent risk factor for isolated SDH, with one cohort showing an eight-fold increased risk of SDH [1]. As the involvement of CAA in SDH pathogenesis is yet to be proven, several pathophysiological mechanisms have been proposed. Traumatic brain injury (TBI) of a lower degree is universally considered the main cause of chronic SDH. The mechanisms of its protracted development are manifold, including post-traumatic neuroinflammation, angiogenesis promoting fragile capillaries creation and proliferation, and fibrinolysis of its clot contents, provoking a response cycle of further inflammation, angiogenesis, and continued microbleedings [6]. Intraoperative findings of leptomeningeal arterial bleeding during SDH evacuation offer a possible arterial origin. Other proposed mechanisms include cerebral atrophy, which may increase susceptibility to bridging veins rupture, and a higher predisposition to falls among patients with CAA [1,4].

This article presents a case series of three patients who experienced acute-onset lobar ICH that was evacuated. Brain biopsy confirmed the diagnosis of CAA. Importantly, an SDH was concurrently observed on head computed tomography (CT) imaging. This case series underscores a potentially under-recognized association between an acute ICH in the context of CAA and coexistent SDH.

## 2. Case Series

### 2.1. Case 1

**Figure 1:**
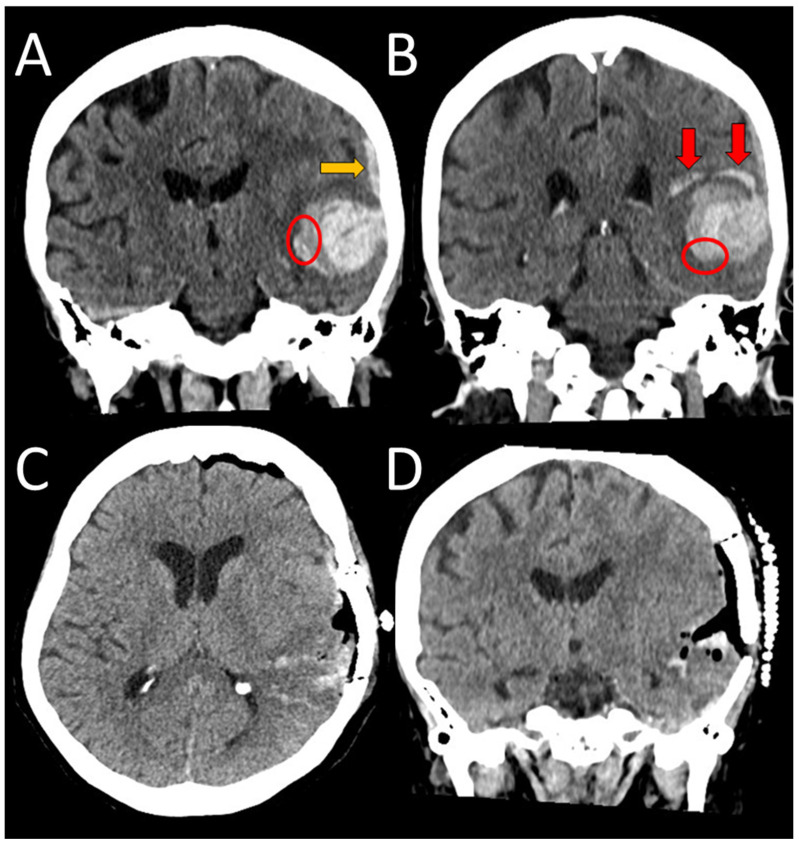
An unenhanced initial CT scan of a spontaneous heterogeneous ICH in the left temporal area (coronal views (**A**,**B**)), measuring 3.7 × 7 × 3 cm. Concomitant subdural hematoma and SAH can be seen (orange arrow), as can finger-like extensions of the bleeding in the brain parenchyma (red arrows). Axial and coronal views after the surgical evacuation ((**C**,**D**), respectively). The ellipse denotes the area of surgical biopsy.

**Figure 2:**
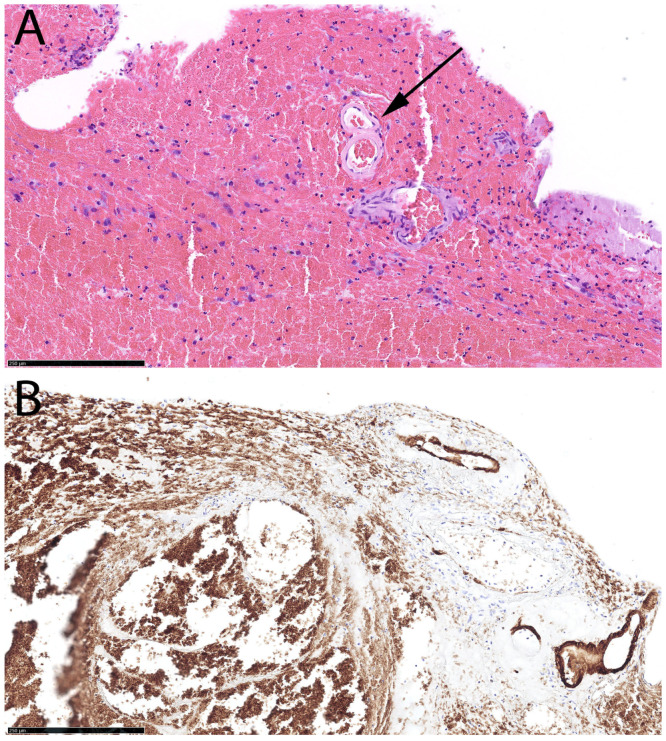
Histopathological findings in Case 1. Focal acellular eosinophilic deposits in the walls of small vessels (arrow) sampled with the hematoma evacuation specimen by Hematoxylin & Eosin stain (**A**). Anti-Aß immunohistochemical stain highlights and confirms the deposits as Aß (**B**). The Aß staining of erythrocytes is non-specific (background).

A 75-year-old right-handed female presented to the neurological emergency department (NED), University Medical Centre (UMC) Ljubljana, in February 2025 with confusion, speech impairment, and headache. Her medical history included hypercholesterolemia, arterial hypertension (AH), and a mood disorder with a premorbid modified Rankin score (mRS) of zero. Neurological examination revealed lip smacking, sensorimotor dysphasia, motor restlessness on the right side, and neglect for tactile stimuli on the right (National Stroke Scale score (NIHSS) 4, mRS 2). Her arterial blood pressure was 175/79 mmHg, and electrocardiogram (ECG) showed sinus rhythm. Laboratory tests, including coagulation studies and tumor markers, were normal, and chest X-ray findings were unremarkable. A head CT scan revealed a heterogeneous lobar ICH in the left parietotemporal lobe, accompanied by an SDH encircling the left cerebral hemisphere, as well as transtentorial herniation (Figure 1A,B). CT angiography was unremarkable, and cerebral venous sinuses and major veins were patent. She was initially treated with levetiracetam due to motor automatisms, which improved after administration but dysphasia remained. Her clinical condition deteriorated later that day, with the development of nausea, vomiting, right-sided spastic hemiparesis, and eye deviation to the left. Follow-up non-contrast head CT revealed hematoma expansion (4.5 × 7 × 4 cm) and increased edema. Urgent ICH hematoma evacuation was performed (Figure 1C,D), and a cortical biopsy was obtained during the same operating session for histopathological analysis, which identified Aβ deposits in the blood vessel walls, confirming CAA (Figure 2). The patient was transferred to the intensive care unit (ICU). Three days after admission, analgosedation was discontinued, revealing sensory-motor dysphasia as the only persistent neurological impairment. On the fifth day of admission, an electroencephalogram was performed, revealing no abnormalities suggestive of epilepsy; however, asymmetry was noted, with a predominance of slow-wave patterns and lower amplitudes in the left hemisphere. She was transferred to a regular ward. Follow-up CT imaging almost one month after admission showed ICH resorption. She became increasingly difficult to manage, initially exhibiting apathic behavior, which later progressed to episodes of excessive agitation, disinhibition, and aggressive outbursts, and eventually followed by psychotic symptoms, necessitating treatment with antidepressants, a mood stabilizer, and an antipsychotic medication.

### 2.2. Case 2

**Figure 3:**
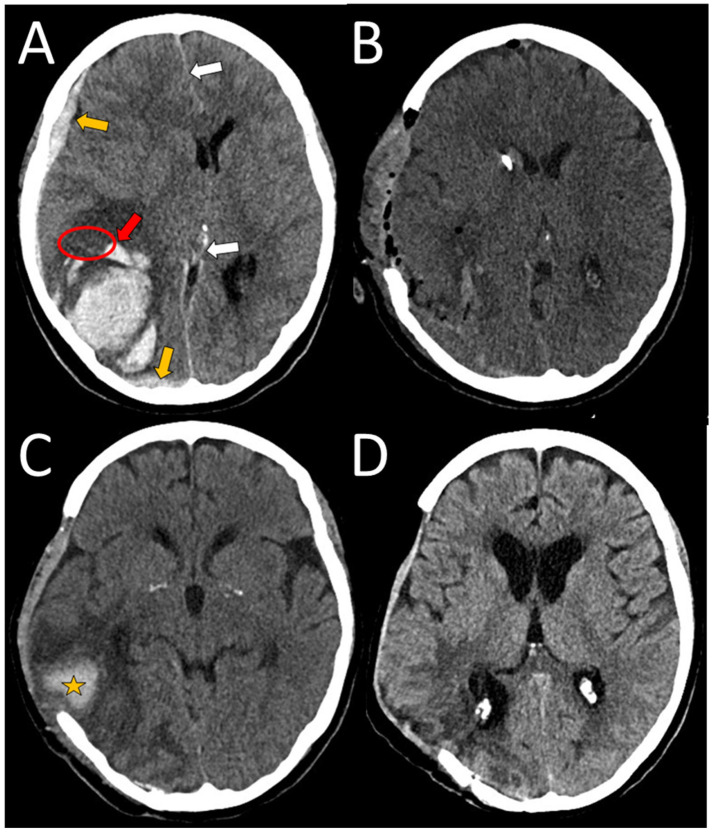
An axial view of a spontaneous ICH in the right occipitoparietal region measuring 6 × 5 × 6 cm, with pronounced brain edema and midline shift (white arrows). The red arrow indicates finger-like extensions in the brain parenchyma. The orange arrows indicate SAH and subdural hematoma (**A**). Postoperative CT scan shows good surgical evacuation of the bleeding, with a removed bone flap (**B**). A week after the second surgery (not shown), a smaller bleeding occurred (orange star), which was treated conservatively (**C**). A few weeks after the second surgery, the brain edema had subsided, and the patient was transferred to a rehabilitation unit. The bone flap has not yet been replaced (**D**). The ellipse denotes the area of surgical biopsy.

**Figure 4:**
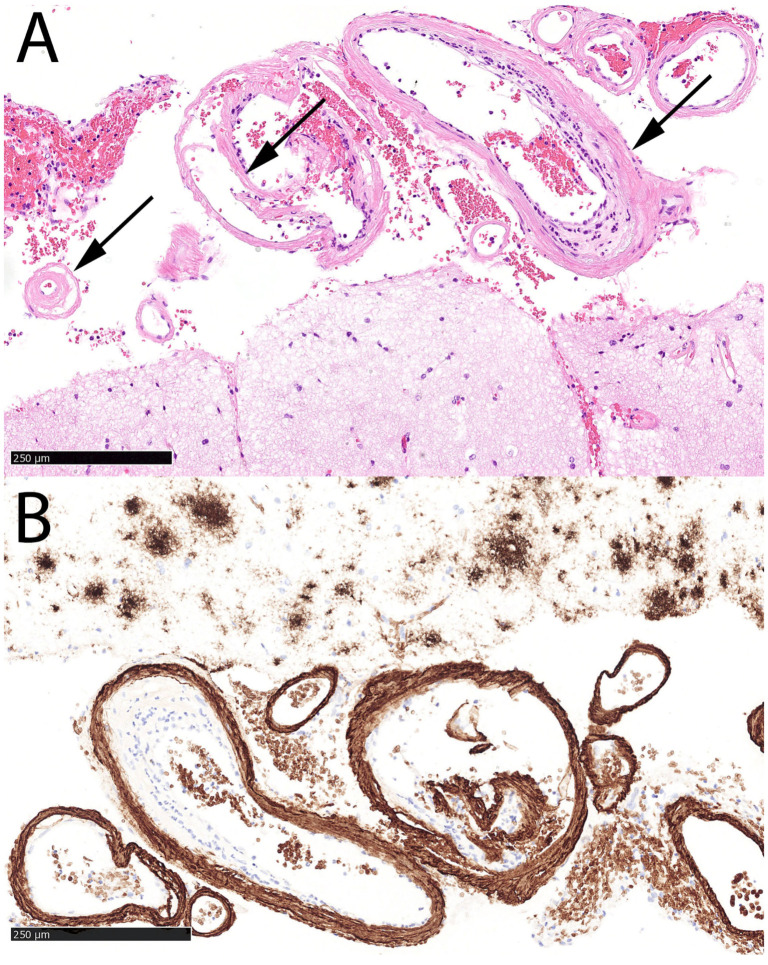
Histopathological findings in Case 2. Hematoxylin & Eosin stain reveals abundant lamellar eosinophilic deposits (arrows) in the walls of leptomeningeal arteries and cortical arterioles (**A**). The deposits correspond to Aß by immunohistochemistry ((**B**), anti-Aß stain). Note also diffuse senile Aß plaques present throughout the neuropil (upper half of the panel) and the non-specific Aß staining of erythrocytes.

A 63-year-old right-handed female, with a history of a severe TBI at an age of 21, without consequences (premorbid mRS 0), presented to NED, UMC Ljubljana, in January 2025 with an acute headache and vomiting, followed by a rapid decline in consciousness to coma (Glasgow coma scale, (GCS) 3). She was analgosedated, intubated, and mechanically ventilated in NED. Her arterial blood pressure was 160/90 mmHg and ECG and laboratory tests were unremarkable. An urgent head CT scan revealed an ICH in the right hemisphere, accompanied by an SDH and transtentorial herniation (Figure 3A). Urgent surgical evacuation of ICH was performed, and the bone flap had been removed and stored for future return (Figure 3B). A cortical biopsy identified Aβ deposits in the blood vessel walls, confirming the CAA (Figure 4). She was subsequently transferred to the ICU. Analgosedation was discontinued on the seventh day after admission. However, 21 days after the initial hemorrhage, her condition deteriorated—she presented with severe left-sided paresis and somnolence. A follow-up CT scan (not shown) revealed a recurrent ICH at the previous hematoma site, measuring 8.3 × 3.2 × 3 cm, with right-sided transtentorial and a more pronounced transcranial herniation. Therefore, a second surgical intervention was performed. After several days, a third hemorrhagic event with a smaller ICH occurred in the region of previously treated ICHs, but she remained asymptomatic (Figure 3C). Over time, her motor deficits significantly improved, but cognitive impairment, including apraxia, disorientation in time, and disturbed visual–spatial functions, was observed. Her rehabilitation continued on an outpatient basis (Figure 3D).

### 2.3. Case 3

**Figure 5:**
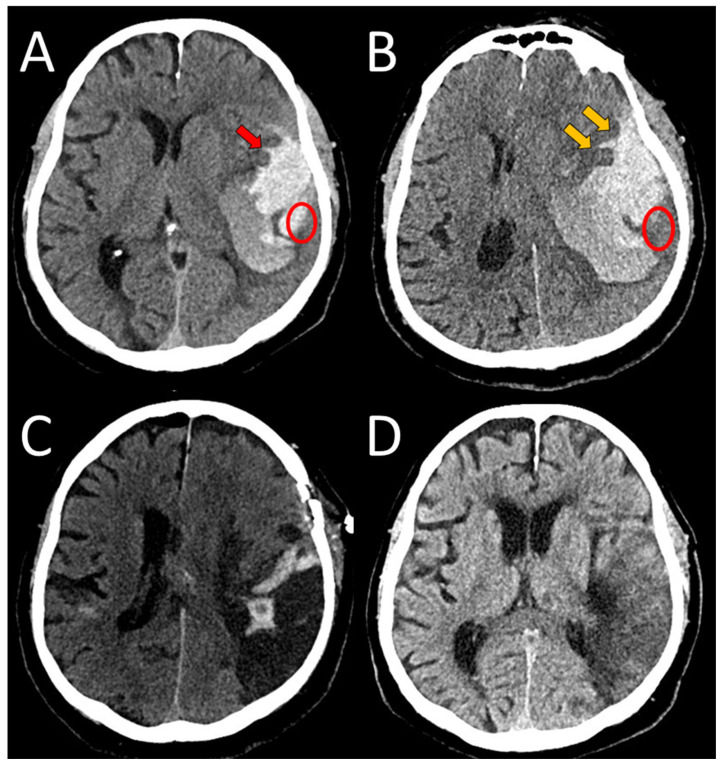
An axial view of a spontaneous ICH in the left parietotemporal region, measuring 4.5 × 7 × 5 cm, with slight brain shift and edema (**A**). A small SAH in the upper part of the Sylvian fissure can be seen (red arrow). The follow-up CT scan after clinical worsening showing a significant ICH progression (**B**), with a more pronounced brain edema and a resultant shift, with further extension of SAH (orange arrows). After a neurosurgical evacuation, a small residual bleeding had been left. The edema and brain shift diminished (**C**). Gradual resorption of the bleeding and improvement of brain edema during the following weeks (**D**) is shown. The ellipse denotes the area of surgical biopsy.

**Figure 6:**
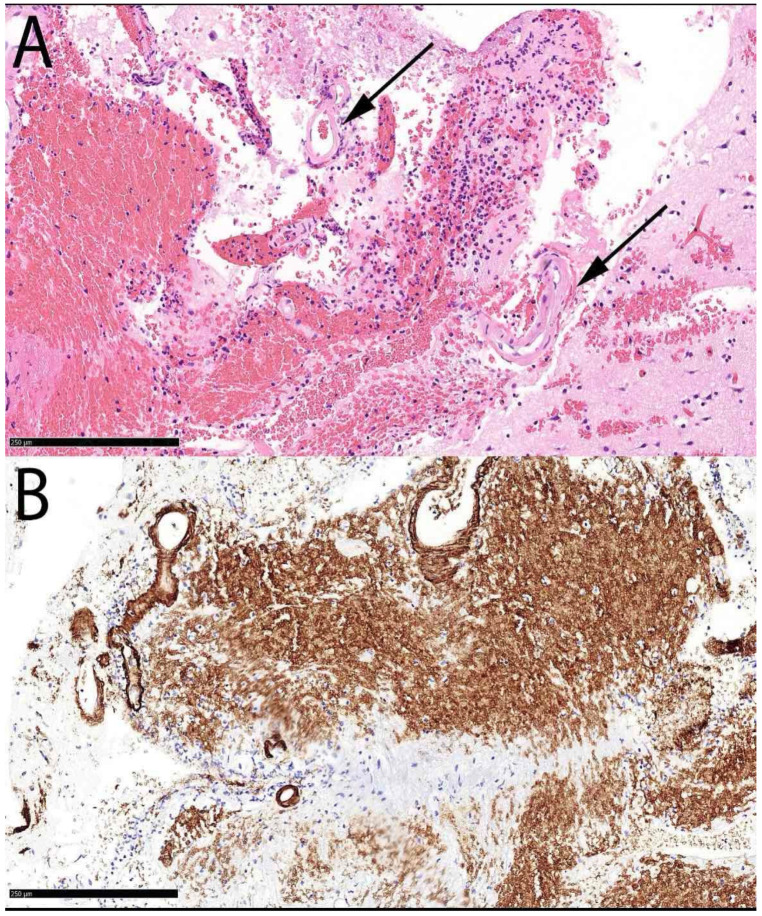
Histopathological findings in Case 3. Lamellar eosinophilic deposits in the walls of small leptomeningeal arteries (arrows) by Hematoxylin & Eosin stain (**A**) that correspond to Aß by immunohistochemistry ((**B**), anti-Aß stain). The Aß staining of erythrocytes is non-specific (background).

A 77-year-old right-handed male with AH, hyperlipidemia, rheumatoid arthritis, and angina pectoris presented to NED, UMC Ljubljana, in March 2025 with acute-onset confusion and speech impairment. Neurological examination revealed somnolence, global aphasia, right-sided hemianopia, deviation of the eyes and head to the left, right-sided facial paresis, and latent paresis of the right arm (NIHSS 15, mRS 5). An initial head CT revealed ICH in the left parietotemporal lobe, an SDH, and a minor SAH in the left Sylvian fissure (Figure 5A). CT angiography was unremarkable. The patient was admitted for intense monitoring, intravenous hydration, and antihypertensive therapy to the stroke unit. Later that day, he developed worsening of consciousness with vomiting. His left pupil became dilated and poorly reactive and he became comatose (GCS 6). A follow-up CT scan revealed ICH expansion with progressive midline shift (Figure 5B). Therefore, urgent surgical intervention with ICH evacuation was performed (Figure 5C). A cortical biopsy revealed Aβ deposits in the blood vessel walls, in line with CAA (Figure 6). Postoperatively, the patient was admitted to the ICU. According to the improvement revealed in the follow-up CT scans (Figure 5D,E), the sedation was discontinued and he was extubated. During ICU stay, he was treated with antibiotics due to pneumonia and urinary tract infection. On day 25, the patient was transferred to the general ward with global aphasia and right-sided hemiplegia. Due to impaired swallowing, a percutaneous endoscopic gastrostomy was performed. During his hospital stay, he developed another urinary tract infection and pneumonia caused by the same organisms previously identified. Neurological improvement was noted, particularly in motor function. At discharge, he exhibited moderate hemiparesis and right-sided hemianopia; however, his speech production and comprehension remained severely impaired (NIHSS 12). He was able to sit unassisted and take a few steps with support (mRS 5). The patient was discharged to outpatient rehabilitation on day 70.

## 3. Discussion

This case series presents three consecutive patients admitted to our medical center over two months with symptomatic acute ICH. All three patients underwent neurosurgical evacuation, during which CAA was confirmed by histopathological analysis. Markedly, in each case, an acute SDH was observed concomitantly with the ICH, despite the absence of any reported head trauma in the preceding months.

To date, the presence of SDH in patients with CAA has been reported only in individual case reports, a retrospective, population-based cohort study involving two large, heterogeneous groups, and two retrospective observational cohort studies. However, no studies have definitively established a causal relationship between SDH and CAA [1].

As noted before, CAA-related intracranial hemorrhages typically include parenchymal microbleeds, macrohemorrhages, and SAH. When non-traumatic SDH occurs in the context of CAA, it is most frequently described as an accompanying hemorrhagic component. Interestingly, a study published in JAMA Neurology demonstrated that SDH may even occur in isolation, without other types of hemorrhages [1]. In a retrospective case–control study conducted by Wenzhou Medical University, patients with CAA-related ICH and accompanying SDH exhibited significantly higher rates of SAH, postoperative rebleeding following hematoma evacuation (similarly to our Case number 2), and worse clinical outcomes. The authors hypothesized that intrasulcal hemorrhage may extend into the subdural space, leading to SDH formation, and suggested that the presence of SAH may be a prerequisite for SDH development in this context [2]. These findings highlight the potential for worse surgical outcomes in patients with CAA-related ICH complicated by SDH and raise important considerations for the management of isolated or concomitant SDH in the context of CAA.

This hypothesis correlates with findings from another study, which showed that SDH and SAH were more frequently seen in patients with ICH and underlying CAA than in patients with ICH unrelated to CAA. Interestingly, this study identified SAH but not SDH as an independent marker associated with CAA-related hemorrhage [7].

There are also reports of SDH as the only radiological finding in CAA. Bruce et al. [8] described a case with histologically proven inflammatory forms of CAA—a more aggressive subtype of CAA characterized by vascular inflammation secondary to amyloid deposition, presenting with isolated SDH [9], which is historically considered a result of head trauma, but recent evidence suggests there are more complex processes involved [6]. What is more, an association between CAA and isolated non-traumatic SDH could form the basis for the exploration of potential genetic associations between CAA and SDH [1]. Although the presence of apolipoprotein E (APOE) ε4 and APOE ε2 alleles has been associated with higher CAA risk and burden [10], APOE genotyping has not been performed in our patients since it is not part of routine management at our institution.

Given the established association between CAA and cognitive decline, including Alzheimer’s disease, an accurate and timely diagnosis may facilitate improved preventive and therapeutic strategies [11].

Moreover, previous medical history of our patients might be significantly associated with their CAA. Preexisting mood disorder in Case 1 might represent one of the key and well-described neuropsychiatric symptoms in CAA, predating intracranial hemorrhage for years or even decades [12]. History of previous TBI in Case 2 might hint at the very pathogenic—posttraumatic—basis of their CAA described in the literature [13]. Rheumatoid arthritis in Case 3, representing a systemic inflammatory condition, might be associated with accelerated accumulation of Aβ in the blood vessels of the brain [14]. Yet, these associations have been described predominantly in inflammatory forms of CAA [15], which were not present in any of our patients.

Anticoagulants pose a significant risk for SDH in particular and intracranial hemorrhage in general in CAA patients [16]. Although their diagnosis of CAA had not been established previously, none of our patients was taking anticoagulants at the time of their bleeding.

In our cases, biopsies of the adjacent brain parenchyma, obtained during neurosurgical evacuation of the hematoma, allowed accelerated diagnosis of CAA, bypassing the need for more time-consuming and invasive diagnostic procedures like lumbar puncture or positron emission tomography–computed tomography. Therefore, a valuable opportunity is suggested—in suspicion of CAA, notably in cases of nontraumatic SDH, brain and/or meningeal biopsy during surgical intervention may be an underutilized diagnostic tool as a routine diagnostic pathway, since magnetic resonance imaging or lumbar puncture are not often pursued and consequently pose a risk of missed or delayed diagnosis.

## 4. Conclusions

We report three patients with histopathologically confirmed CAA who presented acutely with ICH coexisting with SDH, all within two months at a single university medical center. However, the association between CAA and non-traumatic SDH requires larger prospective confirmatory studies. Patients with CAA, who initially present with acute, especially isolated SDH, may be underdiagnosed, consequently leading to delayed identification and missed opportunities for proper risk assessment and management.

## Data Availability

Data are contained within the article.

## Abbreviations

The following abbreviations are used in this manuscript:

Aß	Amyloid β protein
AH	Arterial hypertension
CAA	Cerebral amyloid angiopathy
CT	Computed tomography
ECG	Electrocardiogram
GCS	Glasgow Coma Scale
ICH	Intracerebral hematoma
ICU	Intensive care unit
NED	Neurological emergency department
mRS	Modified Rankin Score
NIHSS	National Institutes of Health Stroke Scale
SAH	Subarachnoid hemorrhage
SDH	Subdural hematoma
TBI	Traumatic brain injury
